# Empirical comparison of deep learning methods for EEG decoding

**DOI:** 10.3389/fnins.2022.1003984

**Published:** 2023-01-10

**Authors:** Iago Henrique de Oliveira, Abner Cardoso Rodrigues

**Affiliations:** Graduate Program in Neuroengineering, Edmond and Lily Safra International Institute of Neuroscience, Santos Dumont Institute, Macaiba, Brazil

**Keywords:** brain machine interface, deep learning, long short term memory, EEG, machine learning

## Abstract

Electroencephalography (EEG) is a technique that can be used in non-invasive brain-machine interface (BMI) systems to register brain electrical activity. The EEG signals are non-linear and non-stationary, making the decoding procedure a complex task. Deep learning techniques have been successfully applied in several research fields, often improving the results compared with traditional approaches. Therefore, it is believed that these techniques can also improve the process of decoding brain signals in BMI systems. In this work, we present the implementation of two deep learning-based decoders and we compared the results with other state of art deep learning methods. The first decoder uses long short-term memory (LSTM) recurrent neural network and the second, entitled EEGNet-LSTM, combines a well-known neural decoder based on convolutional neural networks, called EEGNet, with some LSTM layers. The decoders have been tested using data set 2a from BCI Competition IV, and the results showed that the EEGNet-LSTM decoder has been approximately 23% better than the competition-winning decoder. A Wilcoxon *t*-test showed a significant difference between the two decoders (*Z* = 2.524, *p* = 0.012). The LSTM-based decoder has been approximately 9% higher than the best decoder from the same competition. However, there was no significant difference (*Z* = 1.540, *p* = 0.123). In order to verify the replication of the EEGNet-LSTM decoder on another data, we performed a test with PhysioNet's Physiobank EEG Motor Movement/Imagery dataset. The EEGNet-LSTM presented a higher performance (0.85 accuracy) than the EEGNet (0.82 accuracy). The results of this work can be important for the development of new research, as well as EEG-based BMI systems, which can benefit from the high precision of neural decoders.

## 1. Introduction

Brain-machine interfaces (BMI) aim to translate brain signals into commands that allow the control of machines or computer interfaces (Niemeyer, 2016). One of BMI's best-known paradigms is motor imagery, which refers to the act of imagining a movement without executing it (Mulder, 2007). Thus, if a system can correctly classify the brain signals patterns of motor imagery, patients without motor function can benefit from prostheses, orthoses, exoskeletons, and other neuroprosthetic devices, controlled using thought.

A common method used to record brain electrical activity in non-invasive BMI systems is electroencephalography (EEG) (Bansal and Mahajan, 2019). The EEG signals have complex non-linear properties, low spatial resolution, and are non-stationary (Bhuvaneswari and Kumar, 2015). These limitations make decoding EEG signals a complex and challenging task.

Several statistical methods can be used as neural decoders in BMI systems, such, as an example, Kalman filter (Alarcón-Domínguez, 2017) and linear discriminant analysis (Ahangi et al., 2012). However, as deep learning (DL) is adequate for this purpose, some works are analyzing its feasibility.

Decoders based on convolutional neural networks have been successful in decoding brain signals (Tabar and Halici, 2016; Tang et al., 2017; Lun et al., 2020). EEGNet is a neural decoder based on convolutional neural networks, which was proposed by Lawhern et al. (2018) for the classification of EEG signals showing good performance. Some research has made efforts to improve its performance by combining it with other models, achieving success (Riyad et al., 2020; Wang L. et al., 2020).

The work by Tseng et al. (2019) showed that in some cases, neural decoders based on recurrent neural networks of the long-short term memory (LSTM) type, surpassed traditional decoding methods, such as Kalman filter, wiener filter, and extended Kalman filter.

Besides the relative success in using DL as a decoder in BMI systems, some works are reporting less favorable results, for example, the work of Tseng et al. (2019) employed a LSTM decoder using data from implanted electrodes in three macaques controlling a prosthesis. The results were superior to traditional filter methods for some macaques in some trials but there has not been an overall improvement. These mixed results are due to the large number of hyperparameters that must be evaluated in DL systems, compared to filter methods.

In this work, two neural decoders were implemented. The first neural decoder is based on LSTM, where the characteristics of frequency, time, and space of the signals are extracted separately, through the combination of wavelet packet decomposition (WPD) and common spatial pattern (CSP). This step of pre-processing was chosen based on results presented in the literature (Yang et al., 2012; Feng et al., 2019). The second decoder was called EEGNet-LSTM and combines the features of both models, extracting the characteristics simultaneously with the classification.That decoder is similar to the best decoder implemented by Wang L. et al. (2020), however with differences in the architecture and selection of hyperparameters of the decoder and strategies for data pre-processing.

In both decoders, we exhaustively employed grid search for hyperparameters optimizations, as we believe that is an essential step, to use DL techniques in neural decoding successfully. We detailed all these steps and this may be useful for works that will test DL in BMI in the future.

The neural decoders have been tested with data set 2a from BCI Competition IV (Brunner et al., 2008), which has two motor imaging sessions for four classes (left hand, right hand, both feet, and tongue). To evaluate the performance of the decoders we used two metrics: accuracy and kappa value. We compared the results of the two implemented decoders with each other, as well as with the results obtained by decoders implemented in other works. We found that our deep learning decoders were 23% and 5% better than the best decoder in the competition. We also tested the LSTM-EEG decoder with data from Physiobank EEG Motor Movement/Imagery dataset from PhysioNet, considering two classes of motor imagery (left wrist and right wrist). The EEGNet-LSTM achieved an accuracy of 0.85. The original EEGNet, which was tested by other researchers with the same dataset, showed an accuracy of 0.82.

## 2. Materials and methods

### 2.1. Long short-term memory

The recurrent neural networks long short-term memory (LSTM) can process long data sequences while avoiding gradient vanishing problems (Hochreiter and Schmidhuber, 1997). LSTM networks have a memory cell, called cell state, which is long-term memory, capable of storing information for a long period. Besides, LSTM memory cells have three kinds of gates that control the flow of information, namely: forget gate, input gate and output gate (Du et al., 2021). The update of the memory cell, at each time step, is determined by the following equations (Jiao et al., 2020):


(1) $$\begin{array}{l} f_{t}=\sigma \left(w_{f}\cdot \left[h_{t-1},x_{t}\right]+b_{f}\right) \end{array}$$

The Equation (1) is the forget gate *f*_*t*_, which indicates the information that will be forgotten in the state of the cell. The *w*_*f*_ symbolizes the forget gate weights, *h*_*t*−1_ is the cell's previous output, *x*_*t*_ is the network input, *b*_*f*_ is the bias associated with forget gate.


(2) $$\begin{array}{l} i_{t}=\sigma \left(w_{i}\cdot \left[h_{t-1},x_{t}\right]+b_{i}\right) \end{array}$$

(3) $$\begin{array}{l} {\tilde{C}}_{t}=\sigma \left(w_{c}\cdot \left[h_{t-1},x_{t}\right]+b_{c}\right) \end{array}$$

The input gate *i*_*t*_ is defined by Equation (2), which determines the cell state values that will be updated. The *w*_*i*_ symbolizes the weights and *b*_*i*_ represents the bias associated with the input gate. In Equation (3), ${\tilde{C}}_{t}$ is calculated, generating a vector of candidate values for the state of the cell. These values are calculated using the hyperbolic tangent as activation function. The weights and bias of the cell itself are *w*_*c*_ and *b*_*c*_, respectively.


(4) $$\begin{array}{l} C_{t}=w_{i}*C_{t-1}+i_{t}+{\tilde{C}}_{t} \end{array}$$

In Equation (4), the result of the previous equations is used to update the state of the cell, where *C*_*t*_ is the current state of the cell.


(5) $$\begin{array}{l} o_{t}=\sigma \left(w_{o}\cdot \left[h_{t-1},x_{t}\right]+b_{o}\right) \end{array}$$

(6) $$\begin{array}{l} h_{t}=o_{t}*\tanh\left(C_{t}\right) \end{array}$$

Equation (5) is the output gate *o*_*t*_, which decides the values of the current state of the cell that will be considered in the cell's output. The gate weights are represented by *w*_*o*_ and the bias is *b*_*o*_. The calculation of the output of cell *h*_*t*_ is shown in Equation (6).

### 2.2. EEGNet

EEGNet is a deep learning model based on convolutional neural networks proposed by Lawhern et al. (2018) to be used in classification of EEG signals in BCI systems. This model uses deep and separable convolutions, performing the extraction of signal features and classification at same time.

Table 1 shows the model's architecture, where *C* denotes the number of channels, *T* represents the number of points in time, *F*1 is the number of time filters, *D* is the number of spatial filters, *F*2 represents the number of point filters, *N* is the number of classes and, *LK* is the kernel size of the first layer, also called the temporal convolution length.

**Table 1:** EEGNet architecture based on convolutional block, the output from previous bloc neural networks.

**Block**	**Layer**	**Filters**	**Size**	**Activation**	**Mode**
1	Input				
	Reshape				
	Conv2D	F1	(1, LK)	Linear	Same
	BatchNorm				
	DepthwiseConv2D	D * F1	(C, 1)	Linear	Valid
	BatchNorm				
	Activation			ELU	
	AveragePool2D		(1, 4)		
	Dropout				
2	SeparableConv2D	F2	(1, 16)	Linear	Same
	BatchNorm				
	Activation			ELU	
	AveragePool2D		(1, 8)		
	Dropout				
	Flatten				
Classifier	Dense	N * (F2 * T // 32)		Softmax	

The model has two main blocks and a classification block. In the first block, Conv2D is a convolutional neural network. DepthwiseConv2D is a deep convolution used to learn spatial filters from the temporal convolution performed in the previous layer. The AveragePool2D layers are used in both blocks to reduce the signal-sampling rate. Batch normalization was proposed by Ioffe and Szegedy (2015) to normalize the data for a given layer, in EEGNet it is performed by BatchNorm applied to both blocks. Dropout is the dropout rate and was used in the model to reduce overfitting. In the second block, SeparableConv2D represents separable convolutions, which combine spatial filters in temporal bands. This layer performs a spatial convolution in each input channel and applies a specific convolution to mix the output Chollet (2017). Flatten was used to transform the output of the convolutional layers into a single vector.

In the classification block, the output from previous blocks is transformed by softmax function to perform the multiclass classification.

### 2.3. Wavelet packet decomposition

Wavelets are mathematical functions used to represent data or other functions, at different scales of time and frequency Jiang and Adeli (2004). The Wavelet Packet Decomposition (WPD) is a type of wavelet transform that decomposes a given signal into low-frequency components (approaches) and high-frequency components (details) Faust et al. (2013). Since WPD presents features in both time and frequency domains, this method is useful for parameters extraction from EEG signals, which are non-stationary and have characteristics of multi-scale and randomness Yang et al. (2016). According to Li and Zhou (2016), WPD can be defined recursively as:


(7) $$\{\begin{array}{l} d_{0,0}(t)=x(t),\\ d_{i,2j-1}(t)=\sqrt{2}\sum_{k}h(k)d_{i-1,j}(2t-k),\\ d_{i,2j}(t)=\sqrt{2}\sum_{k}g(k)d_{i-1,j}(2t-k). \end{array}$$

In Equation (7), *x*(*t*) is the original signal, *h*(*k*) is the high-pass filter, *g*(*k*) is the low-pass filter, and *d*_(_*i, j*) are the coefficients of WPD at the *i*−*th* level for the *j*−*th* node (Li and Zhou, 2016).

### 2.4. Common spatial patterns

Common Spatial Patterns (CSP) is a spatial filtering technique widely used for the extraction of EEG features in non-invasive Brain-Computer Interface (BCI) systems (Song and Yoon, 2015). This technique finds spatial filters that will maximize the variance of signals from one class while minimizing the variance from another class, resulting in ideal discriminative features (Cheng et al., 2017). The equations of CSP are presented according to Wang et al. (2005) and Huang et al. (2010):


(8) $$\begin{array}{l} R_{H}=\frac{X_{H}X_{H}{\;}^{T}}{trace\left(X_{H}X_{H}{\;}^{T}\right)}\;R_{F}=\frac{X_{F}X_{F}{\;}^{T}}{trace\left(X_{F}X_{F}{\;}^{T}\right)} \end{array}$$

In Equation (8), assuming that the problem has two classes, *R*_*H*_ and *R*_*F*_ represent the normalized spatial co-variance of each class, *X*_*H*_, and *X*_*F*_ are matrices of the EEG signals of the respective classes, *X*^*T*^ is the transposed from matrix *X* and, *trace*(*A*) is the sum of the diagonal elements of matrix *A*.


(9) $$\begin{array}{l} R=\bar{R_{H}}+\bar{R_{F}}=U_{0}DU_{0}{\;}^{T} \end{array}$$

In Equation (9), the composite spatial co-variance *R* is calculated. $\bar{R_{H}}$ and $\bar{R_{F}}$ are the average normalized covariance, calculated using the average of the co-variance matrices of examples in each class. *U*_0_ is the eigenvector matrix and *D* is the diagonal eigenvalue matrix of *R*.


(10) $$\begin{array}{l} P=D^{-1/2}U_{0}{\;}^{T} \end{array}$$

In Equation (10), the bleaching matrix *P* is calculated, which equalizes the variance in the space defined by *U*_0_.


(11) $$\begin{array}{l} S_{H}=P\bar{R_{H}}P^{T}\;S_{F}=P\bar{R_{F}}P^{T} \end{array}$$

(12) $$\begin{array}{l} S_{H}=UD_{H}U^{T}\;S_{F}=UD_{F}U^{T}\;I=D_{H}+D_{F} \end{array}$$

In Equation (11), the bleaching transformation is applied to $\bar{R_{H}}$ and $\bar{R_{F}}$, obtaining matrices *S*_*H*_ and *S*_*F*_, which share the same eigenvectors. The identity matrix *I* is presented in Equation (12). The eigenvectors that have higher eigenvalues for *S*_*H*_ have smaller eigenvalues for *S*_*F*_, that is, these quantities are inversely proportional, differentiating the classes.


(13) $$\begin{array}{l} W=U^{T}P \end{array}$$

Equation (13) calculates the projection matrix *W*, which allows obtaining non-correlated components of the EEG signals.


(14) $$\begin{array}{l} Z=WX \end{array}$$

In Equation (14), *Z* are the components of the signal *X*, aggregating common and class-specific components.

### 2.5. Description of the datasets

#### 2.5.1. BCI competition IV

This paper used data set 2a from the BCI Competition IV (Brunner et al., 2008), which were registered and made publicly available by Graz University of Technology, located in Austria.

Nine subjects participated in the experiment, which consisted of two motor imaging sessions, held on different days. The objective of the experiment was to imagine four movements, namely, the movement of the left hand, right hand, both feet and, tongue. In each session, 288 attempts at motor imagery have been recorded, with 72 attempts for each movement. It is worth mentioning that each sample of motor imagery has 7.5 s, a time that includes the initial preparation, the realization of motor imagery, and a pause. During the experiment, 22 EEG channels and 3 electro-oculography (EOG) channels were recorded, with sampling rate of 250 Hz. The EEG and EOG electrodes can be consulted in Brunner et al. (2008).

The signals were filtered using a bandpass filter between 0.5 and 100 Hz. In addition, a notch filter was applied at 50 Hz to suppress the noise from the electrical network.

The EOG signals were not recorded correctly for the fourth subject. Therefore, this subject was not considered in this research.

#### 2.5.2. Physiobank EEG motor movement/imagery dataset

To verify the replicability of the best neural decoder developed in this paper, we used the Physiobank EEG Motor Movement/Imagery dataset, freely available from PhysioNet (Goldberger et al., 2000).

One hundred and nine subjects participated in the experiment, which consisted in different tasks of movement execution and motor imagery. However, this paper focuses only on the task of imagining the opening and closing of the left or right wrist, that is, two classes of motor imagery.

During the experiment, a target was displayed on the left or right side of the screen and the subject imagined opening and closing the corresponding fist, until this target disappeared. Over three sessions, subjects performed a total of 45 trials, imagining one of the movements for 4 s. During the execution of the experiment, the brain signals of the subjects were recorded through 64 EEG channels using the international 10–10 system and the BCI2000 toolkit (Schalk et al., 2004), with a sampling rate of 160 Hz.

### 2.6. Implementation of the LSTM decoder

**Figure 1:**
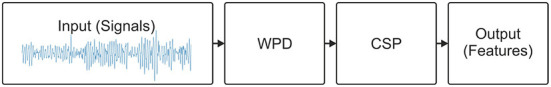
Features extraction steps for the LSTM decoder.

The extraction of features is an important step in the classification of EEG signals (Amin et al., 2017). Some works have used the combination of WPD and CSP to extract features and have achieved better results compared to the use of CSP only (Yang et al., 2012; Feng et al., 2019). In this research, we use a combination of WPD and CSP to extract important resources for the LSTM-based neural decoder. Initially, the preprocessed EEG signals are used as input to the four-level WPD, obtaining the coefficients of the wavelet transform. Then, the extraction of features from these coefficients was performed through the CSP. This process is shown in Figure 1.

**Figure 2:**
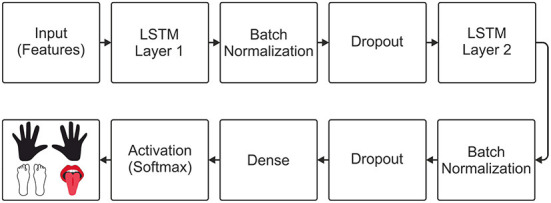
Architecture of the LSTM neural decoder.

For the implementation of the neural decoders, the python programming language (Python, 2020) and the keras library (Keras, 2020) were used. For the implementation of the LSTM-based decoder, an input layer was initially added to the model, allowing the input of features obtained through WPD and CSP. Inspired by the model that obtained the best performance in the work by Tseng et al. (2019), two LSTM layers were included in the model. After each LSTM layer, batch normalization was applied to normalize the outputs, and the dropout to avoid overfitting the model. Finally, a dense layer was inserted with the number of units equal to the number of classes in the data set used, and a softmax activation function was added, allowing multiclass classification. The architecture of the LSTM decoder is shown in Figure 2.

### 2.7. Implementation of the EEGNet-LSTM decoder

**Figure 3:**
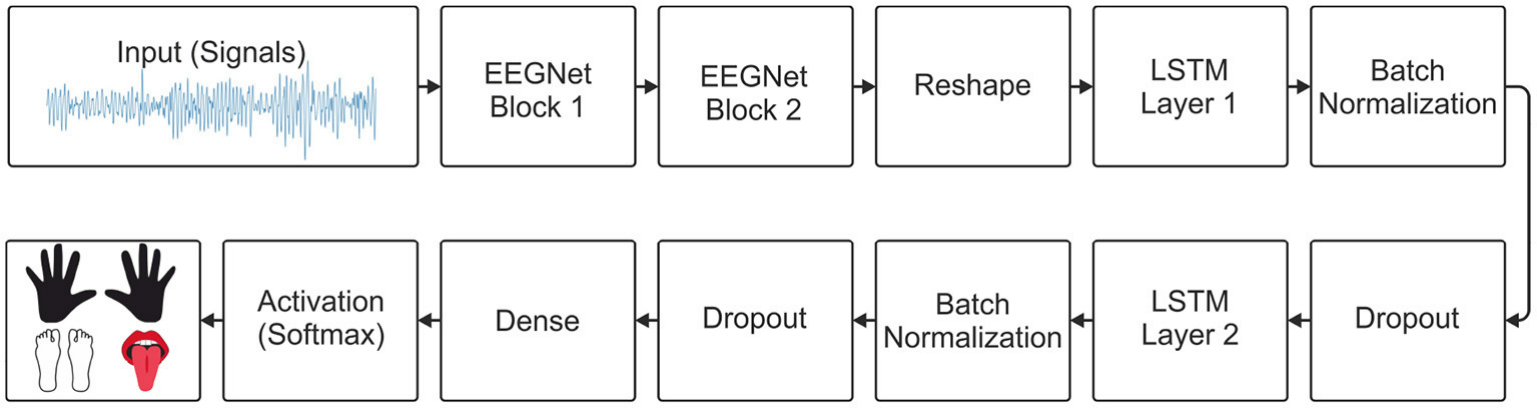
Architecture of the EEGNet-LSTM neural decoder.

For the implementation of the model proposed here, the two main blocks of the EEGNet model were used, as specified by Lawhern et al. (2018). Then, a layer was used to reshape the output of the last block of the EEGNet model and connect its output to the 2 LSTM layers. After each LSTM layer, batch normalization and dropout were used. A dense layer with the number of units equal to the number of classes and a softmax activation function for multiclass classification. The architecture of the EEGNet-LSTM decoder is shown in Figure 3.

### 2.8. Data pre-processing

#### 2.8.1. Data preprocessing from BCI competition IV dataset 2a

As the preprocessing step is very important for the good performance of neural decoders, we tested four different bandpass filters. The first one was a bandpass filter between 0.5 and 100 Hz, the second was between 8 and 13 Hz (mu rhythm), the third was between 15 and 30 Hz (beta rhythm), and the fourth was between 8 and 30 Hz (mu and beta rhythms). The frequency ranges referring to mu and beta rhythms were defined according to Bear et al. (2020).

The labels of the four classes of motor imagery (left hand, right hand, both feet and tongue) were coded using one-hot encoding, respectively, in four-dimensional vectors: {[1, 0, 0, 0], [0, 1, 0, 0], [0, 0, 1, 0], [0, 0, 0, 1]}.

Then, only 4 s of each sample were selected. Among the 7.5 s of each sample, only signals registered between 2 and 6 s were considered, during which time a suggestion of motor imagery was presented on the screen and was performed by the subject. Finally, the data was normalized between –1 and 1.

#### 2.8.2. Data preprocessing from the Physiobank EEG Motor Movement/Imagery dataset from PhysioNet

The same bandpass filters applied in the BCI Competition IV dataset 2a were applied to Physiobank data. As Wang X. et al. (2020) did not report the application of any filter, we also tested the use of raw signals, without any filtering. Additionally, we also tested a bandpass filter between 0.5 and 45 Hz.

Each motor imagery attempt has a duration of 4 s. However, we select only the first 3 s, as suggested by Wang X. et al. (2020). Thus, for each motor imagery attempt we have 480 samples for each of the 64 EEG channels.

Next, we separate the training and test data according to Wang X. et al. (2020). Data from subjects 1 (S001) to 84 (S084) were used as a training set. Subjects 85 (S085) to 109 (S109) were used as a test set.

In their paper, Wang X. et al. (2020) mentioned the removal of four subjects, but did not specify which ones. Thus, we removed subjects 88 (S088), 92 (S092), 100 (S100) and 104 (S104) because they were damaged, according to Varsehi and Firoozabadi (2021) and Fan et al. (2021).

The labels of the two classes of motor imagery (left wrist and right wrist) were coded using one-hot coding. Finally, we scaled brain signals between –1 and 1.

### 2.9. Hyperparameter optimization

#### 2.9.1. Hyperparameter optimization of LSTM and EEGNet-LSTM decoders for BCI Competition IV dataset 2a

The hyperparameter optimization was performed using the data from first subject of the data set. Then, we freeze the parameters and used them to train the models for the other subjects.

The data set has two sessions, one used for training and the other for testing. For each configuration test, accuracy and value of the kappa coefficient (Cohen, 1960) were recorded, so that the best configuration was identified. Table 2 presents the common hyperparameters between the LSTM based decoder and the EEGNet plus LSTM decoder.

**Table 2:** Common hyperparameters of both models.

**Hyperparameter**	**Values**
Optimizer	Adam, RMSprop, SGD
Learning rate	0.0001, 0.001, 0.01
Batch size	32, 64, 128
Regularization of L2	0.1, 0.2, 0.3
Dropout	0.1, 0.2, 0.3
Epochs	100, 200, 300, 400, 500, 1,000

#### 2.9.2. Adjusting hyperparameters of the LSTM decoder

Initially, the LSTM-based decoder was trained with 32 neurons in each layer, with different configurations shown in Table 2. The best configuration was maintained based on the highest accuracy and kappa value, and the decoder was tested with different amounts of neurons, according to Table 3.

**Table 3:** Hyperparameters specific to the LSTM decoder.

**Hyperparameter**	**Values**
Neurons layer one	32, 64, 128, 256
Neurons layer two	32, 64, 128, 256

Based on these tests, it was possible to identify an ideal configuration for the decoder and this configuration was used for the other subjects.

#### 2.9.3. Adjusting hyperparameters of the EEGNet-LSTM decoder

The EEGNet-LSTM decoder has been trained with LK = 64, F1 = 8, *D* = 2 and F2 = 16, which are standard for EEGNet. In the LSTM layers, the same number of neurons obtained through tests with the LSTM-based decoder was maintained. Then, all configurations in Table 2 were tested, obtaining the best configuration for the model. Soon after, the hyperparameters of Table 4 were tested to identify the best configuration of the specific hyperparameters of EEGNet.

**Table 4:** Hyperparameters specific to the EEGNet decoder.

**Hyperparameter**	**Values**
LK	16, 32, 64, 128
F1	4, 6, 8, 16
F2	4, 6, 8, 16
D	1, 2, 4, 6

#### 2.9.4. Hyperparameter optimization of the EEGNet-LSTM decoder for the Physiobank EEG motor movement/imagery dataset

To adjust the EEGNet-LSTM decoder hyperparameters for the Physiobank EEG Moviment/Imagery dataset, we performed the same steps performed for the previous dataset. The only difference is that for this dataset, we used a 30% of the data from the training set to use as a test during hyperparamer search, instead of using the first subject as we did with BCI data.

## 3. Results

### 3.1. Best hyperparameters for the implemented decoders

#### 3.1.1. Best hyperparameters for decoders LSTM and EEGNet-LSTM using BCI Competition IV dataset 2a

After automatically making all possible combinations of the hyperparameters, the best settings for the two neural decoders were obtained. Table 5 presents the configurations (common between the two decoders) that provided the best results. Regarding the specific hyperparameters of the LSTM-based neural decoder, it presented better results with 32 neurons in each layer. For the EEGNet-LSTM neural decoder, the specific EEGNet configurations that generated the best results were F1 = 16, *D* = 6, F2 = 16 and FK = 16.

**Table 5:** Best selected hyperparameters for the decoders considering the data set 2a from BCI Competition IV.

**Hyperparameter**	**LSTM**	**EEGNet-LSTM**
Optimizer	Adam	Adam
Learning rate	0.0001	0.001
Batch size	64	32
Regularization of L2	0.2	0.2
Dropout	0.2	0.2
Epochs	200	400

Regarding the filtering configuration, the best results were obtained through the standard filtering of the data set, that is, between 0.5 and 100 Hz. The best filtering and the best hyperparameters were used to decode the signals of all subjects.

#### 3.1.2. Best hyperparameters for the EEGNet-LSTM decoder using the physiobank EEG motor movement/imagery dataset from PhysioNet

Table 6 presents the large search result, which returned the best combination of hyperparameters for the EEGNet-LSTM decoder, when using the Physiobank EEG Movement/Imagery dataset.

**Table 6:** Best selected hyperparameters for the EEGNet-LSTM decoder considering the data set Physiobank EEG Motion/Imagery from PhysioNet.

**Hyperparameter**	**EEGNet-LSTM**
F1	16
D	4
F2	16
FK	16
Optimizer	Adam
Learning rate	0.01
Batch size	128
Regularization of L2	0.2
Dropout	0.2
Epochs	200

The best results were achieved after applying a bandpass filter between 0.5 and 45 Hz. This filtering and the best hyperparameters were used to train the EEGNet-LSTM and decode the data from the test dataset.

### 3.2. Comparison between the implemented decoders using BCI competition IV dataset 2a

Using the best configurations, the neural decoders were trained with the data from one session and tested with the data from another session, making it possible to evaluate their performance, in the classification of four classes of motor imagery.

**Figure 4:**
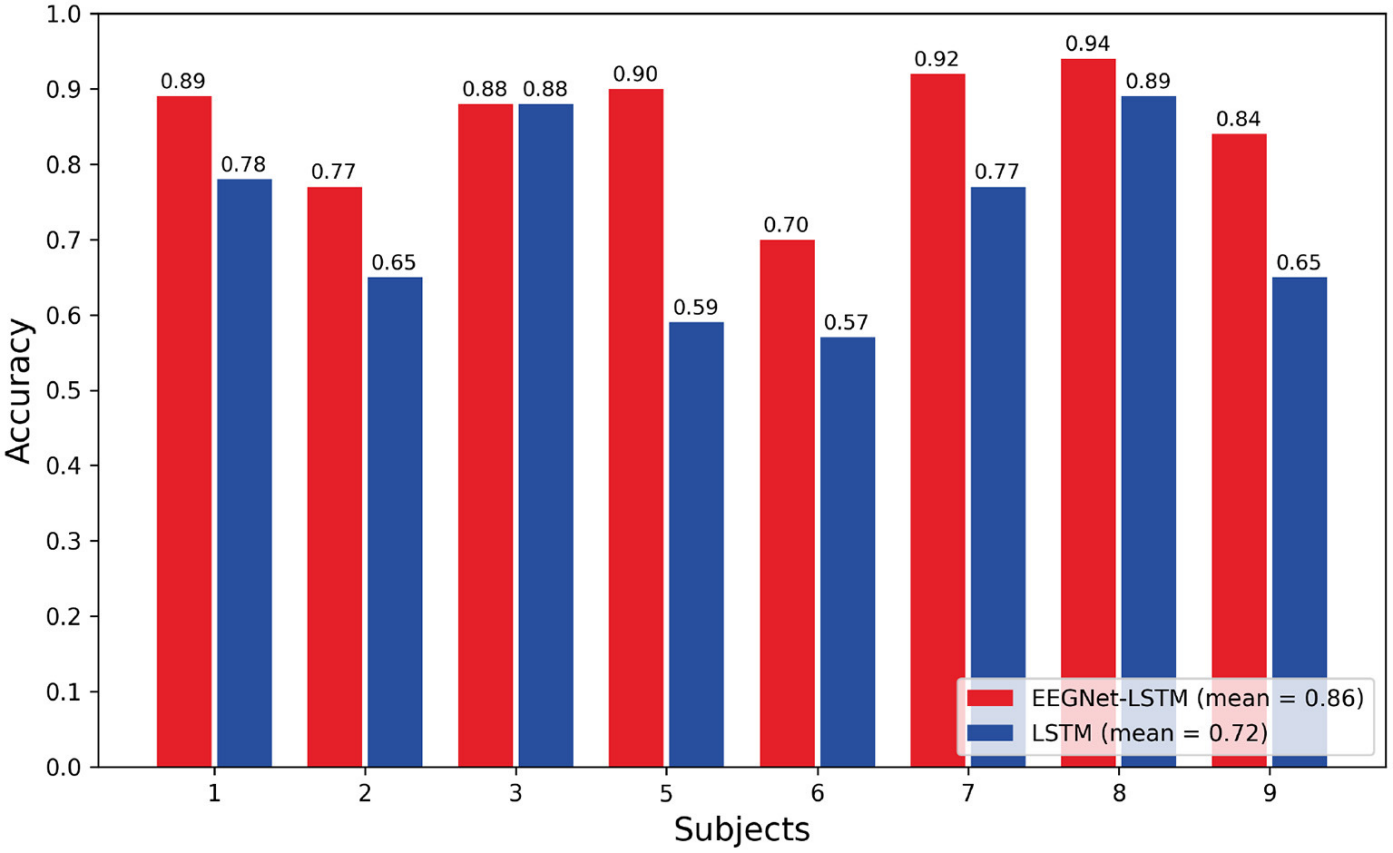
Comparison of the accuracy obtained through the decoders.

Figure 4 shows the comparison graph between the accuracy of each subject, obtained through the two neural decoders implemented: LSTM and EEGNet-LSTM. The second decoder obtained a better result for all subjects, except subject 3, in which the two decoders presented equal accuracy. The average accuracy for the EEGNet-LSTM neural decoder was 0.86 and for the LSTM decoder, it was 0.72. Therefore, the average accuracy of the EEGNet-LSTM neural decoder was about 14% higher than the average accuracy of the LSTM decoder. A Wilcoxon *t*-test showed a significant difference between the accuracy of the neural decoders (*Z* = 2.366, *p* = 0.018).

**Figure 5:**
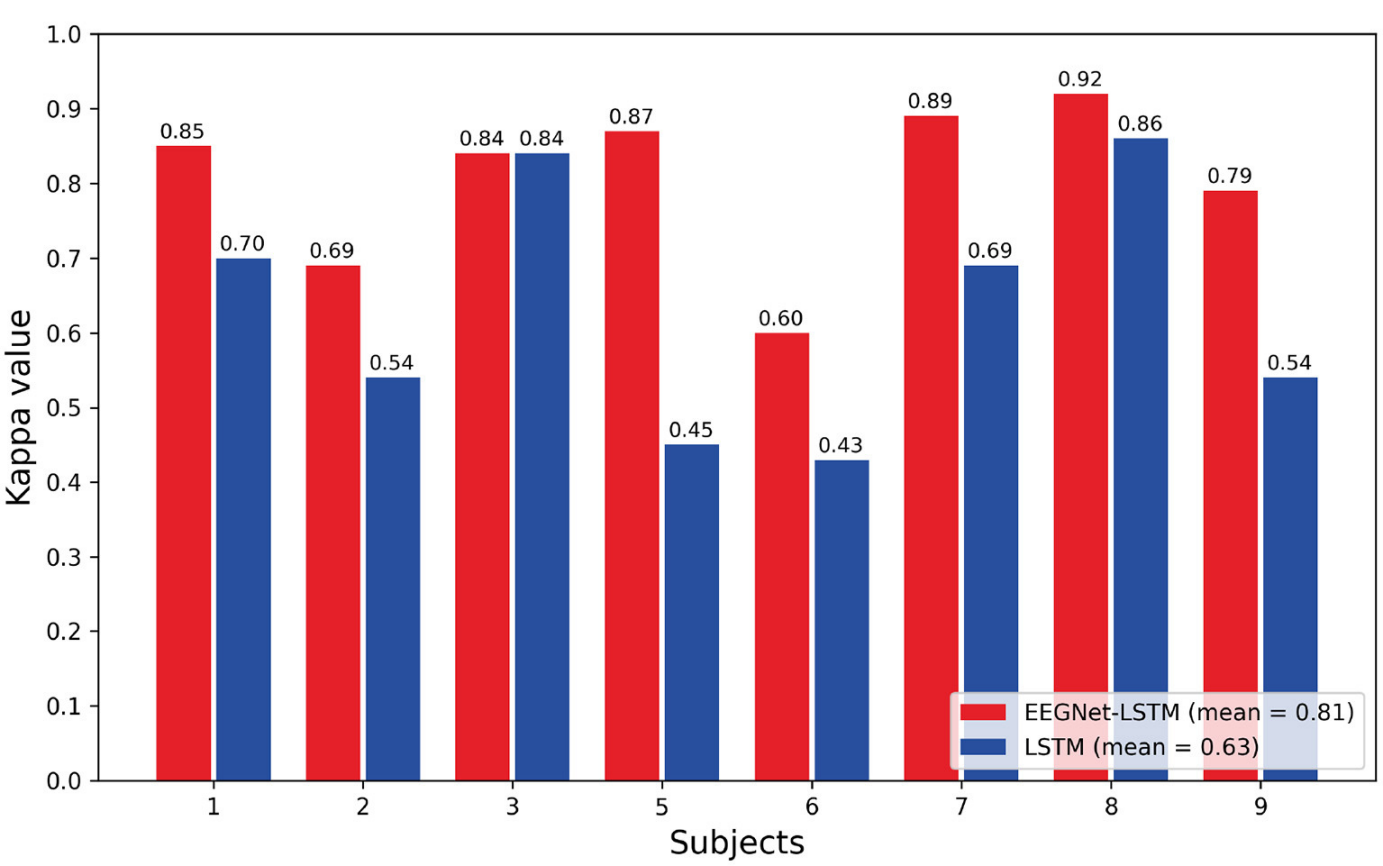
Comparison of the kappa values obtained through the decoders.

Figure 5 shows the comparison bar plot between the kappa values obtained for each subject and the average value for each decoder. The LSTM decoder presented the average kappa value equal to 0.63 and the decoder that combines EEGNet and LSTM resulted in an average kappa value equal to 0.81. According to the interpretation suggested by Landis and Koch (1977), the first decoder presented a strong agreement, and the second, an almost perfect agreement. The average kappa value obtained using the EEGNet-LSTM neural decoder was approximately 18% higher than the average kappa value achieved by the LSTM decoder. A Wilcoxon *t*-test indicated a significant difference between the kappa values of the two decoders (*Z* = 2.371, *p* = 0.018).

### 3.3. Comparison with other results published in the literature that made use of the BCI competition IV dataset 2a

Table 7 presents a comparison between the kappa values obtained through the decoders implemented in the present work and the kappa values achieved by decoders developed in other works.

**Table 7:** Comparison with the kappa values achieved by other studies.

**Methods**	**Subjects**								**Mean**	**Standard deviation**	**p value (Wilcoxon)**	
	**1**	**2**	**3**	**5**	**6**	**7**	**8**	**9**			**EEGNet-LSTM**	**LSTM**
EEGNet-LSTM (Proposed method)	0.85	0.69	0.84	0.87	0.60	0.89	0.92	0.79	0.81	0.109	-	0.018
LSTM (Proposed method)	0.70	0.54	0.84	0.45	0.43	0.69	0.86	0.54	0.63	0.167	0.018	-
FBCSP e NBPW (Ang et al., 2012)	0.68	0.42	0.75	0.40	0.27	0.77	0.75	0.61	0.58	0.192	0.012	0.123
Incep-EEGNet (Riyad et al., 2020)	0.71	0.37	0.87	0.48	0.47	0.88	0.76	0.79	0.67	0.198	0.043	0.326
SCCRNN (Wang L. et al., 2020)	0.77	0.38	0.75	0.54	0.47	0.76	0.78	0.70	0.64	0.157	0.012	0.888
FBSF-TSCNN (Chen et al., 2020)	0.81	0.47	0.84	0.32	0.43	0.77	0.76	0.74	0.64	0.202	0.018	0.753

Based on the average kappa value, the EEGNet-LSTM neural decoder was about 23% higher than the method proposed by Ang et al. (2012), first place in the BCI Competition IV, which used the filter bank common spatial pattern (FBCSP) for the extraction of characteristics and the naive bayesian Parzen window (NBPW) for the classification. A Wilcoxon *t*-test indicated a significant difference between the two decoders (*Z* = 2.524, *p* = 0.012). The EEGNet-LSTM decoder provided a result approximately 14% higher than the Incep-EEGNet developed by Riyad et al. (2020). There was a statistically significant difference (*Z* = 2.028, *p* = 0.043). This decoder was about 17% higher than the decoder entitled series compact convolutional recurrent neural network (SCCRNN), proposed by Wang L. et al. (2020). The test indicated a significant difference (*Z* = 2.527, *p* = 0.012). The average kappa value was also approximately 17% above the filter bank spatial filtering and temporal-spatial convolutional neural network (FBSF-TSCNN), presented by Chen et al. (2020). There was a statistically significant difference (*Z* = 2.527, *p* = 0.018).

The LSTM-based decoder achieved a result about 5% higher than the result obtained using the Ang et al. (2012) method. However, there was no significant difference (*Z* = 1.540, *p* = 0.123). The LSTM neural decoder presented a result approximately 4% lower than the result obtained through the Riyad et al. (2020) method. However, there was no significant difference (*Z* = 0.981, *p* = 0.326). The LSTM decoder gave a result about 2% below the result of Wang L. et al. (2020). However, there was no significant difference (*Z* = 0.141, *p* = 0.888). This decoder obtained a result approximately 1% lower than the result achieved by the Chen et al. (2020) method. However, there was no statistically significant difference (*Z* = 0.314, *p* = 0.753).

### 3.4. EEGNet-LSTM decoder performance with Physiobank EEG Motor movement/imagery dataset

Considering the two classes of motor imagery (left wrist and right wrist) from the Physiobank EEG Motion/Imagery dataset from PhysioNet, the EEGNet-LSTM decoder presented an accuracy of 0.85 in the test set. Using the same dataset, Wang X. et al. (2020) tested EEGNet and achieved an accuracy of 0.82. Using Filter Bank Common Spatial Pattern (FBCSP) and Support Vector Machine (SVM), Handiru and Prasad (2016) achieved approximately 0.64 accuracy.

## 4. Discussion

This work aimed to develop accurate neural decoders. The EEGNet-LSTM and LSTM decoders achieved, respectively, accuracies equal to 0.86 and 0.72. The high hit rate suggests that the decoders developed have great potential for future applications in EEG-based BMI systems.

In this work, a combination of WPD and CSP was performed to extract the characteristics of the signals, for the LSTM-based decoder. According to Yang et al. (2012), this combination provides better results compared to the use of CSP only, due to the time and frequency characteristics of the WPD. Using WPD, the signals were represented in different scales of frequency and time, and the spatial characteristics were extracted through the CSP.

LSTM-type networks can store information for long periods in their memory (Tseng et al., 2019). Therefore, these networks allowed the retention of information of imagined movements and, provided good performance in the decoding of the signals. The decoder surpassed the best result of BCI competition IV, but the results were slightly lower than the results obtained by other researchers, who used decoders that made use of convolutional neural networks (Chen et al., 2020; Riyad et al., 2020; Wang L. et al., 2020).

Although the average kappa value was slightly worse compared to decoders that used convolutional neural networks, for some specific subjects the LSTM decoder provided greater or equal results. The average kappa value provided by the LSTM decoder was approximately 2% lower than the decoders of Wang L. et al. (2020) and Chen et al. (2020). Therefore, the percentage difference was very small.

The other decoder implemented in this paper, called EEGNet-LSTM, combined the features of the two models, aiming to obtain a better performance. Wang L. et al. (2020) developed some neural decoders and the best was SCCRNN, similar to the decoder implemented here, since both combine EEGNet with two LSTM layers. The frequency and spatial characteristics can be extracted by CNN, and the temporal characteristics can be extracted by the LSTM layers.

However, unlike the network model implemented in this paper, Wang L. et al. (2020) used a fully connected layer before the first LSTM layer. In the EEGNet-LSTM decoder, implemented in the present paper, only the two main EEGNet blocks were used and the characteristics extracted through these blocks were passed directly to the LSTM layers. After each LSTM layer, batch normalization and dropout layers were also added to avoid overfitting the model. The use of these layers was not mentioned by Wang L. et al. (2020).

In addition, after testing different frequency ranges of the signals, bandwidth filtering between 0.5 and 100 Hz was considered, which generated the best results. In the research by [3], a bandpass filter between 4 and 35 Hz was applied. In the present work, the labels of the imagined movements were encoded in binary vectors, using the one-hot encoding method, being another difference that can influence the results. Other works used in the comparison also did not mention the use of this technique.

The EEGNet-LSTM decoder implemented in this research, surpassed the results of current decoders (Chen et al., 2020; Riyad et al., 2020), including the best decoder proposed by Wang L. et al. (2020), which has an architecture similar to the decoder implemented in this work. The additional layers, the exhaustive selection of hyperparameters, the strategies used in the pre-processing of the signals, and the fact of passing the characteristics extracted by the EEGNet blocks, directly to the LSTM layers, is what must be behind the better performance.

The combination of WPD and CSP allows the extraction of time-frequency and space features. However, using these methods, the extraction of characteristics and, the classification through LSTM decoder, are steps performed separately. According to Wang L. et al. (2020), performing the feature extraction and classification, separately, may not provide ideal results, and it is recommended to perform the extraction and classification stage together, since the extraction of characteristics can be adjusted automatically, based on the classification. The EEGNet-LSTM decoder performs the extraction of characteristics and, the classification, together, providing better results in comparison to the decoder based on LSTM, with the extraction of resources through WPD and CSP.

Wang X. et al. (2020) demonstrated that there is a reduction in performance metrics of the model, as the number of motor imagery classes increases. We noticed that the EEGNet-LSTM presents a similar result to the original EEGNet in a simpler problem, involving two classes of motor imagery from the Physiobank EEG Motor Movement/Imagery dataset. However, we noticed that in a more complex problem, involving the four classes of motor imagery from the BCI Competition IV dataset 2a, the EEGNet-LSTM presented significantly higher results than the Incep-EEGNet, an improved version of the EEGNet. Therefore, additional LSTM layers increase the hit rate, especially in more complex problems. The EEGNet-LSTM presented satisfactory results for two different datasets, one simpler and the other more complex. Considering that brain-machine interface systems can be used to control multiple actuators coupled to prostheses, orthoses and exoskeletons, the model presented is useful, as it maintains a high success rate in a more difficult problem.

## 5. Conclusion

Through this work, it was possible to observe better results, when feature extraction and classification are performed together. It was noted that the selection of hyperparameters and the pre-processing of the data are essential for the good performance of the decoders. It was also possible to notice that when combined with other models, LSTM-type networks have the potential to improve results, mainly due to their temporal capacity. The EEGNet-LSTM neural decoder showed satisfactory results for two different datasets, proving the potential for replicability and ability to maintain a high rate of success in simpler problems (two classes of motor imagery) and more complex (four classes of motor imagery). Given the good results compared to competing neural decoders, the EEGNet-LSTM decoder implemented in this research can be a good alternative for accurate decoding of EEG signals in BMI systems. Therefore, it can serve as a starting point for the development of future works.

## Data availability statement

Publicly available datasets were analyzed in this study. This data can be found here: https://www.bbci.de/competition/iv.

## Ethics statement

Ethical review and approval were not required for the study on human participants in accordance with the local legislation and institutional requirements. The patients/participants provided their written informed consent to participate in this study.

## Author contributions

IO implemented the algorithms and wrote the manuscript. AR conceived and supervised the project. Both authors contributed to the article and approved the submitted version.
